# Retinoblastoma research trends from 1980 to 2023: a 44-year bibliometric study

**DOI:** 10.3389/fonc.2025.1549387

**Published:** 2025-05-28

**Authors:** Yun Zhao, Yining Wang, Yuchuan Wang, Jiagen Li, Hongxun Li, Hong Zhao

**Affiliations:** ^1^ Department of Ophthalmology, Tianjin Eye Hospital, Tianjin, China; ^2^ Tianjin Key Laboratory of Ophthalmology and Visual Science, Tianjin Eye Institute, Tianjin, China; ^3^ Nankai University Affiliated Eye Hospital, Nankai University, Tianjin, China; ^4^ Clinical College of Ophthalmology, Tianjin Medical University, Tianjin, China

**Keywords:** retinoblastoma, publication trends, bibliometric analysis, intravitreal chemotherapy, CiteSpace

## Abstract

**Background:**

Retinoblastoma (RB) is the most common intraocular malignant tumor in children. It not only seriously threatens patients’ vision but also endangers their lives if not treated in time. Our objective is to analyze research trends in the RB field and compare contributions from different countries, institutions and authors.

**Methods:**

We extracted all RB-related publications published from 1980 to 2023 from the Web of Science database and applied VOSviewer, R software, Bibliometrix software, Origin 2024 and CiteSpace to review the publication data, analyze the publication trends, and visualize the relevant data. In this study, the research papers on RB published in the past 44 years were classified by year, country/region, institution/university, journal, author and keywords to reveal the research hotspots and development trends in this field.

**Results:**

A total of 4156 papers on RB were identified from 1980 to 2023. In 1980, only 13 papers were published, yet by 2023, 237 papers had been published. These publications were contributed by 351 research institutes from 68 countries/regions. The United States ranked first with 1662 papers, accounting for 39.99% of the total number of publications on RB research. A total of 539 RB research papers were published in China, ranking second. India, Canada and Germany ranked third, fourth and fifth, with 377, 277 and 221 publications, respectively. Thomas Jefferson University published the most research papers on RB, with 166 published papers, accounting for 3.99% of all publications. The top three journals contributing to this field were *Invest Ophth Vis Sci*, the *British Journal of Ophthalmology* and *Ophthalmology*. Liquid biopsy, intra-arterial chemotherapy and intravitreal chemotherapy are the most frequently used keywords in the field.

**Conclusion:**

Over the past 44 years, the United States, China, India, Canada and Germany have led the field of research on RB. Many renowned research institutions and ophthalmologists have made important contributions to RB research and will continue to lead this research direction. Liquid biopsy, intra-arterial chemotherapy and intravitreal chemotherapy are potential hotspots for RB research in the future.

## Introduction

Retinoblastoma (RB) is the most common intraocular malignancy in children, particularly those younger than 3 years (1, 2). The reported global incidence is 1 in 16000 to 18000 live births. The development of RB is thought to be related to the suppression of rapid and uncontrolled cell growth by the retinoblastoma gene (RB1) on chromosome 13 (3). Worldwide, approximately 25–35% of cases impact both eyes, with diagnoses for bilateral cases typically established before the age of 12 months and for unilateral cases around the age of 24 months (4). RB is a serious threat to children’s vision and survival. With the continuous development of ophthalmic treatment technology, the treatment of RB has progressed from enucleation surgery and radiotherapy to systemic chemotherapy and *in situ* chemotherapy (5). The survival rates of RB are currently reported to be greater than 95% in developed countries and much lower in low-income developing countries (6).

Over the past four decades, the treatment of RB has made great progress, with significant improvements in eye preservation and survival rates (7). Bibliometrics is the best method for revealing the research trends, hot issues and future development directions of a particular research field through statistical analysis of the publication times, authors, institutions, topics and number of citations in the literature during a certain period (8). The bibliometric analysis provides a powerful, objective method to map the evolution of RB research. Through quantitative analysis of publications, citations, and partnerships, bibliometric analysis reveals research trends, hot topics, and emerging topics in RB that may not be evident in individual studies. Here, we conducted a bibliometric examination of studies on RB from January 1980 to December 2023 by the distribution of annual publications, journals, countries, institutions, authors, keyword co-occurrence, and burst terms. Compared to previous studies, this is the first bibliometrics study of RB to cover a 44-year span (1980-2023), providing a longer and more comprehensive time frame than earlier studies. Our aim was to identify RB research trends and hotspots, offering references for future investigations.

## Materials and methods

### Data acquisition

**Figure 1:**
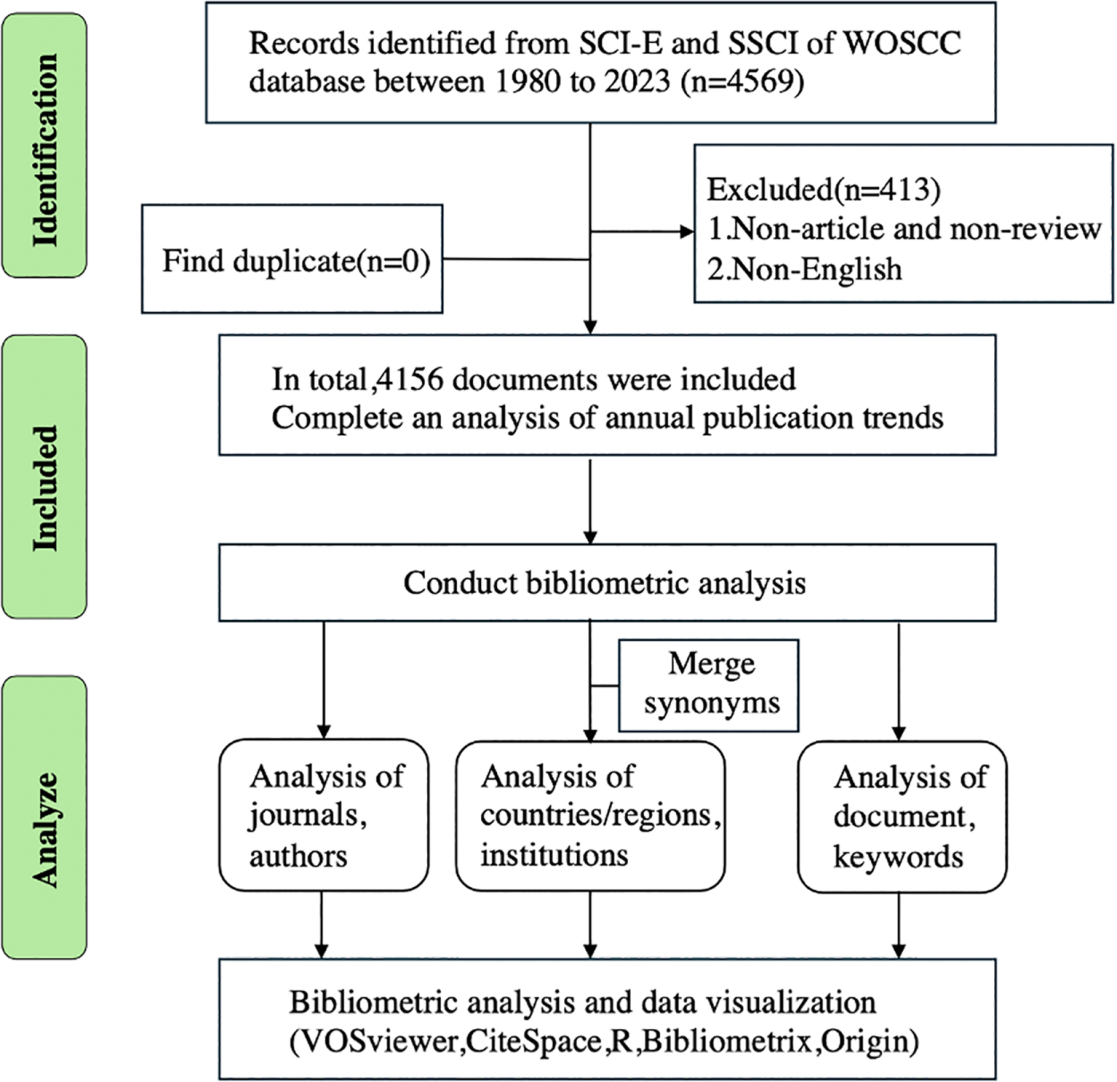
Flow diagram of RB researches inclusion process.

The Web of Science Core Collection (WOSCC) is widely used in bibliometric research. We conducted all searches for RB on March 23, 2021, at the WOSCC for publication retrieval and analysis to avoid bias associated with daily database updates. The search dates for this study were from January 1, 1980, to December 31, 2023, and the language of the literature was limited to English. The document type was limited to “article” and “review”. The data used were “plain text” formats with complete records downloaded from the WOSCC database and contained all recorded and cited references. The final search formula was (TI = (retinoblastoma) AND TS= (eye OR ocular OR oculus OR optical OR ophthalmic OR ophthalmology OR intraocular OR optic OR retinal OR retina)) AND Language=English. A total of 4569 publications, comprising 3698 articles, 458 reviews, 356 conference papers, 135 editorial materials, 111 letters, 24 notes, 11 errata, and 224 others, were included. Finally, 4156 publications (3698 original articles and 458 reviews) were included. The literature screening process and research process are shown in **Figure 1**.

### Bibliometrics and visualization analysis

We obtain publication numbers, countries and regions, authors, citations and H-Index values for these papers through the WOSCC database. The papers included in this study were downloaded and analyzed by researchers, and bibliometric analysis and statistical analysis were performed using CiteSpace (version 6.2. R6), VOSviewer (version 1.6.19), Bibliometrix and Origin 2024.

## Results

### Overview of publications on RB

**Figure 2:**
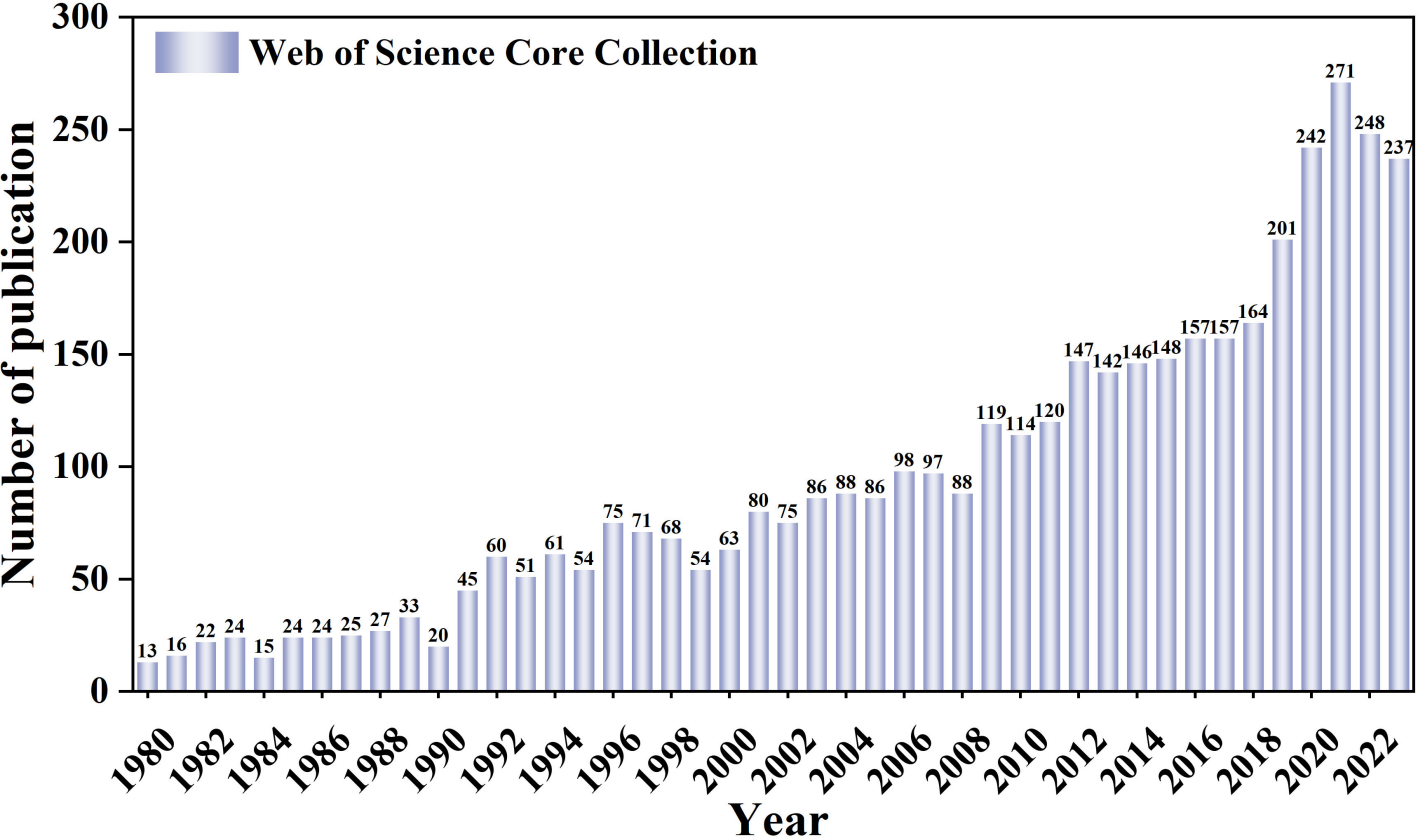
Number of publications per year.

In this study, we identified 4156 papers on RB in the past 44 years for bibliometric analysis. We searched the WOSCC database and found only 13 papers on RB in 1980; however, the number of papers increased to 237 to 2023. The number of publications peaked at 271 in 2021 and decreased in the following two years, which may be due to a decrease in research due to the impact of the COVID-19 pandemic. Since the beginning of the 21st century, the number of published research articles on RB has increased rapidly, especially in the last decade. The number of RB-related papers published in 2023 is more than 18 times that published in 1980. The annual number and change trend of RB research papers published from 1980 to 2023 are shown in **Figure 2**.

### Geographical distribution of published countries/regions

**Figure 3:**
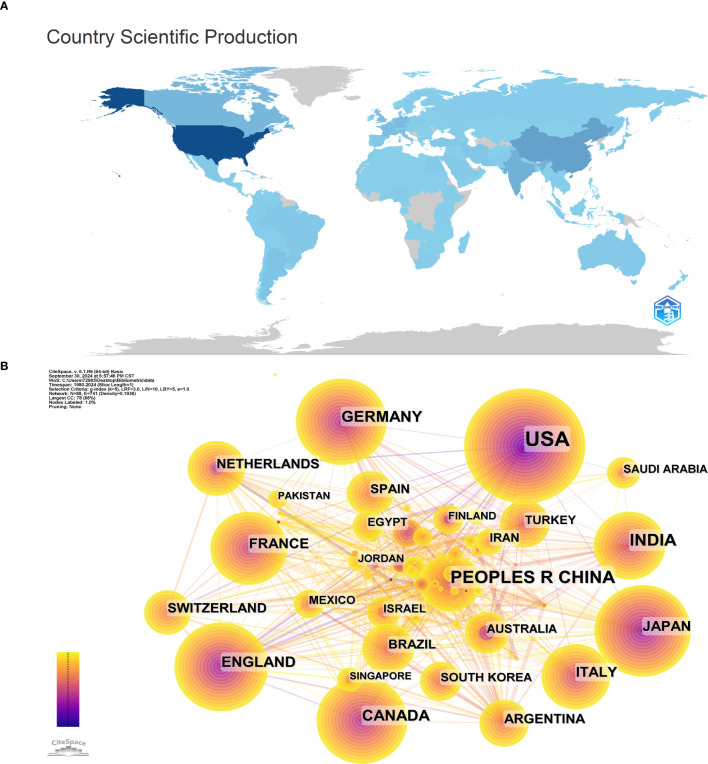
**(A)** Geographic distribution map based on the total publications. **(B)** Network visualization analysis of research countries (regions).

A total of 68 countries/regions were retrieved in this study over the past 44 years. **Figure 3A** shows a geographic distribution map of the total publications. The United States ranked first with 1662 papers, more than the number of papers published in the next five countries combined. The United States accounted for 39.99% of the total number of publications on RB research. A total of 539 RB research papers were published in China, ranking second. India, Canada and Germany ranked third, fourth and fifth, with 377, 277 and 221 publications, respectively. **Figure 3B** shows the country/region collaboration network created via the coauthor analysis method. The size of the nodes in **Figure 3B** represents the number of articles published in each country, and the links between the nodes represent collaboration. The strength of the connection reflects the strength of the cooperation.

### Geographical distribution of published research institutions/universities of RB

**Figure 4:**
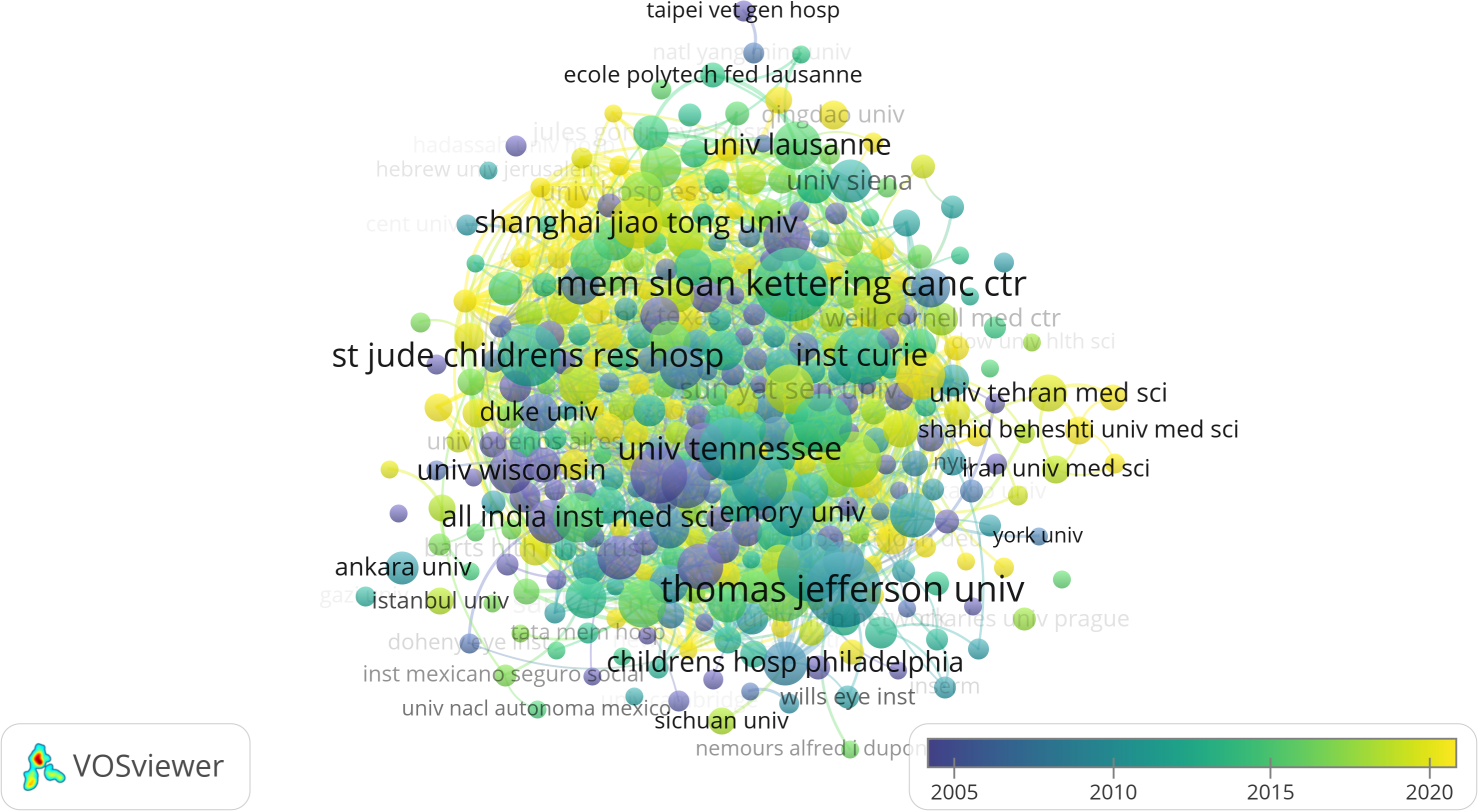
Network visualization analysis of research institutions/universities.

A total of 351 research institutions/universities published papers on RB from 1980 to 2023. Thomas Jefferson University published the most research papers on RB, with 166 published papers, accounting for 3.99% of all publications, far more than other institutions or universities. The second, third, fourth and fifth papers were from the Memorial Sloan Kettering Cancer Center, the University of Toronto, St. Jude Children’s Research Hospital, and the University of Tennessee, with 158 (3.8%), 127 (3.06%), 106 (2.55%) and 103 (2.48%) papers, respectively. A research institution collaboration network, illustrated in **Figure 4**, was created using the coauthor analysis method.

### Journal distribution of RB publications

**Figure 5:**
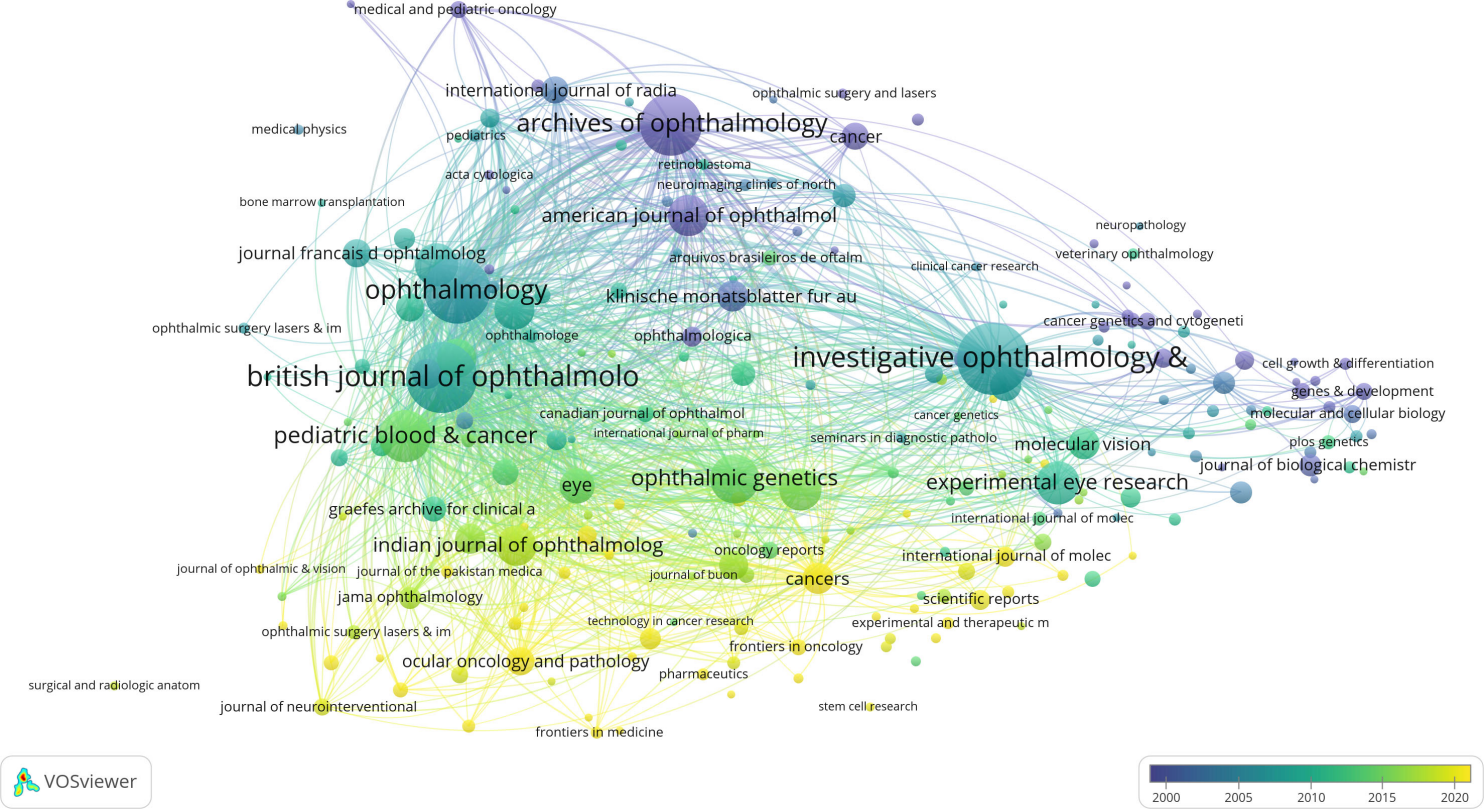
Citation network of journals in RB research.

In the past 44 years, 4156 publications on RB research were obtained from 210 journals. Among all the journals, the most numerous and influential research papers on RB were published in *Invest Ophth Vis Sci*, with 142 publications, followed by the *British Journal of Ophthalmology* (n=139) and *Ophthalmology* (n=124). The top three journals in terms of the number of publications are all ophthalmology journals with international influence, and these journals are sponsored by countries in Europe and the Americas. The citation network of journals in RB research is shown in **Figure 5**.

### Authors publishing research on RB

**Figure 6:**
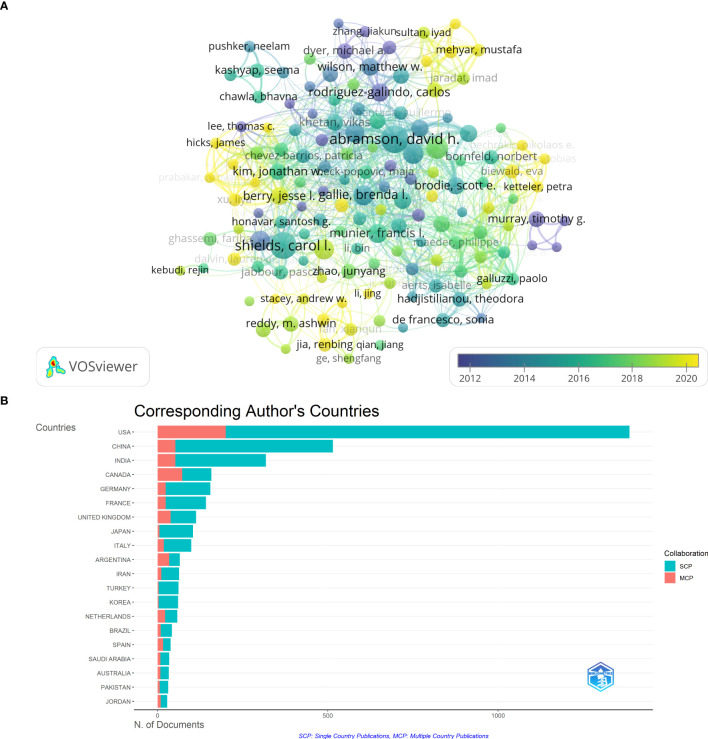
**(A)** Citation network of cooperative research of the authors. **(B)** Countries of the corresponding authors in RB research, showing the number of papers from single-country publication (SCP) or multiple-country publications (MCP).

A total of 647 papers were published by the top 10 authors, accounting for 15.6% of all literature in the field (**Table 1**; **Figure 6A**). Abramson DH of the Memorial Sloan Kettering Cancer Center of the United States published 117 papers related to RB, ranking first in the number of publications. Shield CL of Thomas Jefferson University in the United States ranked second, with 110 publications. Francis JH of the Memorial Sloan-Kettering Cancer Center of the United States ranked third, with 65 papers published. According to the influence of the top 10 authors, Shield CL has the 1st H-Index, whereas Shield JA has the 2nd highest. **Figure 6B** shows the country distribution of corresponding authors of research papers on RB over 44 years, with the number of collaborative research papers from multiple countries in red and the number of research papers from a single country in blue. The United States ranks first in terms of the total number of corresponding authors, the number of multinational collaborative papers, and the number of papers in a single country. China ranks second in terms of the number of publications by corresponding authors, and the proportion of multinational collaborative papers is lower than that in Canada.

**Table 1:** The top 10 authors according to the number of publications.

Rank	Author	H index	Total citations	N	Country
1	Abramson DH	57	4743	117	United States
2	Shield CL	95	4681	110	United States
3	Francis JH	31	1453	65	United States
4	Dunkel IJ	52	2482	56	United States
5	Gallie BL	67	2725	54	Canada
6	Kaliki S	39	1012	54	India
7	Shields JA	83	2949	54	United States
8	Chantada GL	41	2094	48	Argentina
9	Munier FI	42	1904	47	United States
10	Rodriguez-Galindo C	72	2100	42	United States

### Analysis of keywords and focuses of RB research

**Figure 7:**
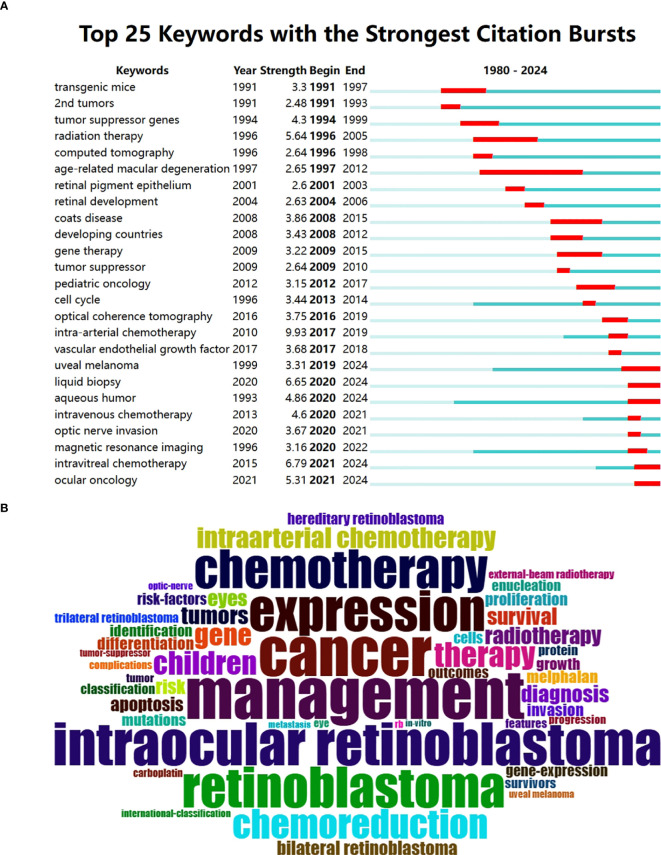
**(A)** Top 25 keywords with the strongest citation bursts in RB research. **(B)** Word cloud based on the most frequent keywords of articles retrieved with the search. The size of the words is proportional to their frequency.

Keywords can reflect the research focus and development trend of articles in the field of RB research. We used CiteSpace to generate the top 25 keywords with the strongest citation bursts, and **Figure 7A** shows the generated keyword burst graph. Among the top 25 keywords, those with the strongest bursts were “intra-arterial chemotherapy” and “intravitreal chemotherapy”. The keywords with the longest duration of use were age-related macular degeneration and radiation therapy. Among these keywords, uveal melanoma, liquid biopsy, aqueous humor and ocular oncology have become more prevalent in recent years. A word cloud of the keywords used in RB research is shown in **Figure 7B**. The most prominent keywords are management, cancer, intraocular retinoblastoma, expression, retinoblastoma, chemotherapy, chemoreduction, therapy, children, and genes.

### Analysis of topics trends of RB research

**Figure 8:**
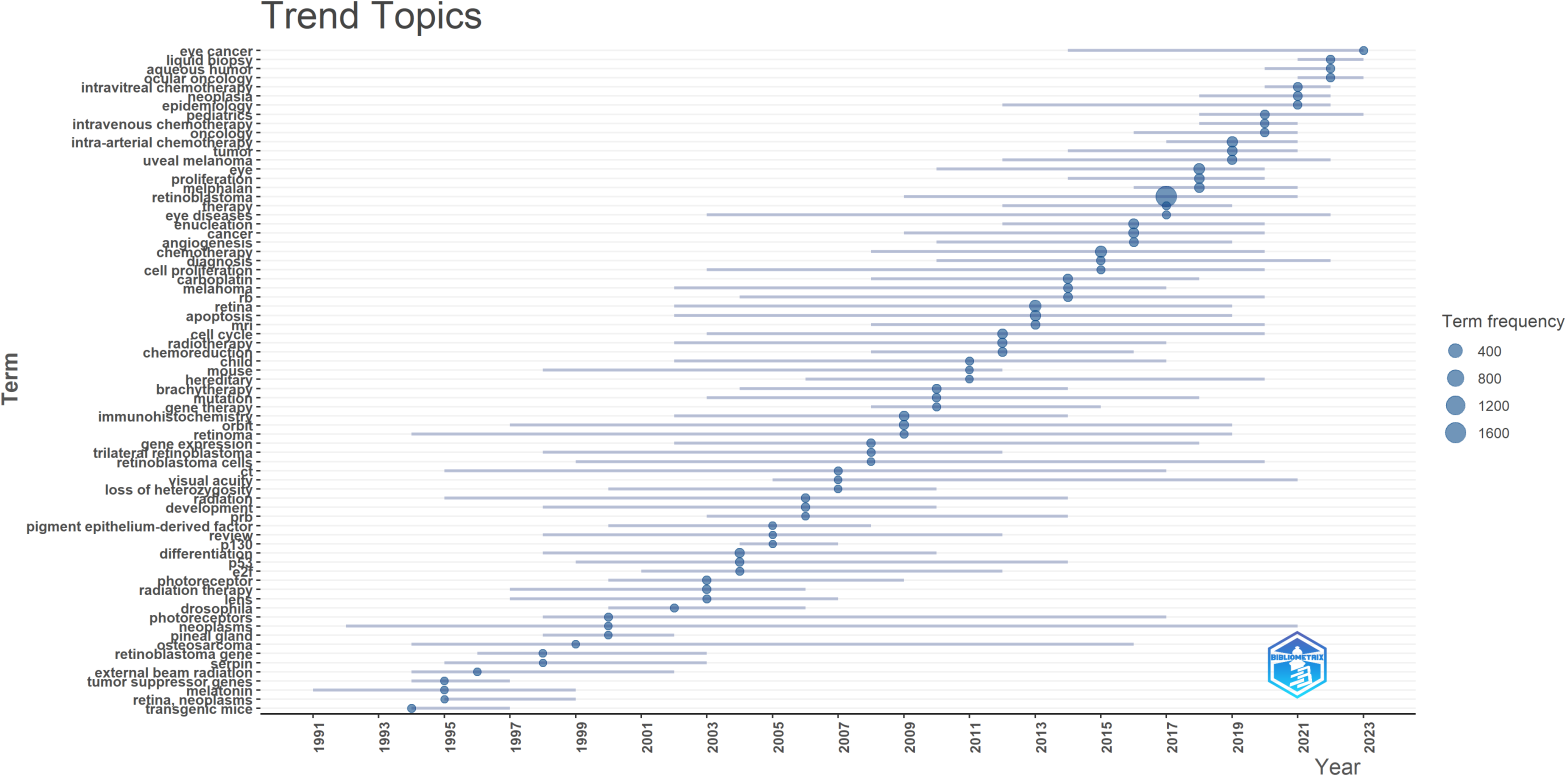
Trend topics of research of RB from 1980 to 2023.

Trend analysis of the keywords revealed that from 1980 to 2000, research focused on transgenic mice, melatonin, tumor suppressor genes, external beam radiation genes and retinoblastoma genes. From 2001 to 2020, researchers began to focus on chemotherapy, apoptosis, immunohistochemistry and the cell cycle. Notably, since 2020, liquid biopsy, intravitreal chemotherapy and aqueous humor have become popular research topics in the field of RB. **Figure 8** shows the trend topics of research on RB from 1980 to 2023.

## Discussion

Through bibliometric analysis, this study presents the global research status of RB in the past 40 years and identifies the main knowledge areas and emerging trends in RB research. We found that between 1980 and 2023, the United States, China, India, Canada and Germany were the five countries that published the most papers on RB research. We also found that four of the top five authors were from the United States. This indicates that the United States is a global leader in RB research. However, China and India have developed rapidly in this area of research, with the number of papers published increasing rapidly annually. There are many possible reasons for this global trend in RB research. Previous studies revealed significant differences in the incidence of RB based on sex, ethnicity, and infections due to poor sanitation (9). However, in recent years, studies have suggested that the incidence of RB is similar worldwide (10). Therefore, we speculate that the differences in the incidence of RB may not be the main factor for the differences in research levels between different regions of the world. Through this paper’s bibliometric analysis, we can provide some possible reasons for this phenomenon. On the one hand, these countries, with many published articles, have high-level scientific research and clinical institutions, many highly educated clinicians and researchers, advanced equipment and diagnosis and treatment technology, sufficient scientific research funds, and a high-quality national clinical research registration system (11, 12). On the other hand, the population base may be an important factor affecting the level of RB research (13). The top three countries, the United States, China and India, have relatively large populations and many RB patients. It is beneficial to carry out clinical research and drug development with a large sample size (14).Moreover, the size of the population of hundreds of millions or even more than one billion people has significant genetic diversity, and it is possible to carry out genetic studies on RB in different ethnic groups, even within a country (15). We emphasize the crucial importance of collaborative research on rare tumors among diverse countries and regions (16). Academic exchanges between various clinical research institutions play a pivotal role in maintaining a balanced global development of RB research (17). The substantial number of multinational collaborative research papers published in the United States and Canada can be attributed to their strong academic exchange traditions, active researcher collaborations, shared language and cultural backgrounds, robust academic organizations and mutually recognized clinical research registries (18, 19). Comparing the trends of authors and international cooperation at different times, we find that in the early stage, researchers and cooperation between European and American countries dominated, but in recent years, research cooperation in developing countries has increased significantly, especially in China, India and other countries.

A range of ophthalmic clinical research institutions and researchers have published many RB-related articles (20–22). This reflects the fact that RB treatment is currently concentrated in several experienced ocular oncology centers (23). A lack of experience in the treatment of RB and the ability to correctly diagnose ocular tumors may delay the treatment of patients and affect their survival time (24, 25). The treatment of RB may be limited by the technical conditions of different treatment centers, and local chemotherapy, such as transocular arterial chemotherapy, is often chosen by ophthalmology centers in cooperation with interventional radiology centers, whereas pediatric cancer centers tend to choose more comprehensive systemic chemotherapy (26). Thus, as researchers have suggested, it is necessary to increase awareness of RB among public and ocular physicians. For patients with RB with suspicious lesions, receiving treatment at an experienced ocular oncology center may help achieve better tumor control (27, 28). Notably, journals of ophthalmology, such as *Invest Ophth Vis Sci*, the *British Journal of Ophthalmology* and *Ophthalmology*, were the primary journals publishing research on RB. Therefore, future papers in the field of RB research are more likely to be published in these journals.

Over the past 44 years, researchers from different national and regional research institutions have published results on the clinicopathological characteristics and treatment methods of RB, which have been widely cited. In 1987, Lee et al. reported the susceptibility gene of RB and described the diversity of genetic loci and mutations of this gene, which has been cited 1316 times (29). RB gene research contributes to prenatal testing, genetic counseling and research on the pathogenesis and treatment of RB. In 1992, Lee published another paper in Nature on the important role of the RB gene in nerve and hematopoiesis in mice, which was cited 1167 times (30). In 1994, Morgenbesser et al. reported that the retinoblastoma tumor suppressor gene is involved in negative growth regulation, the induction of differentiation, and the inhibition of cellular transformation; this article was published in *Nature* and was cited 580 times (31). At this stage, the genetic mutation of RB and possible targeted therapies became a hot research topic, and many highly cited articles were published. In 2006, Laurie et al. provided evidence that the p53 pathway is inactivated in RB and that MDMX may be a specific chemotherapeutic target for treating RB (32). These genetic studies help elucidate the pathogenesis of RB and provide a theoretical basis for developing innovative treatment methods. Moreover, the highly cited papers published in well-known journals, such as Nature and Science, also increase the research heat of RB in ophthalmology. In 2012, Deimaras et al. published a review of retinoblastoma in the prestigious medical journal Lancet, which summarized the research results of the stage at that time. RB was the first tumor to draw attention to the genetic causes of cancer. Studies at the gene level have enabled genetic counseling and genetic screening for RB. Moreover, multidisciplinary clinical research has reduced the mortality of patients. In the same year (33), Zhang et al. proposed a new treatment for retinoblastoma through genomic and epigenetic studies and published it in Nature (34). They reported that the proto-oncogene SYK is upregulated in retinoblastoma and is required for tumor cell survival. Targeting SYK with small molecule inhibitors induces retinoblastoma tumor cell death *in vitro* and *in vivo*. RB may develop rapidly as a result of epigenetic dysregulation of key cancer pathways, directly or indirectly, caused by the loss of RB1. Rushlow et al. described the genetic and clinical features of RB with RB1 mutations (35). As more is known about RB, treatments are improving. Patients’ demand for improved quality of life and appearance continues to rise. Currently, fewer enucleation surgeries are being performed, and the focus of treatment research has gradually shifted to intravenous, intra-arterial and intravitreal chemotherapy. With the development of clinical research and the release of new clinical guidelines, the application of intra-arterial chemotherapy for treating different stages of RB has expanded, becoming one of the primary treatment modalities for intraocular RB worldwide (36, 37).

We reviewed the literature and found previously published bibliometric analyses on RB (38–40), but the bibliometric analyses included in this study over a period of up to 44 years provide unique insights into the analysis of trends in RB research. Gu et al. (38) conducted a bibliometric analysis of the research literature on RB from 2001 to 2021. The ranking of the top three countries in terms of the number of published articles was the same as that of this study, but the research time span was shorter. They concluded that potential future research hotspots include intravitreal, intraarterial and intravenous chemotherapy. Obviously, with the research and development in recent years, this hot topic has changed. The conclusions of the bibliometric studies conducted by Aykut et al. (39) and Shemesh et al. (40) both emphasized the importance of collaboration for achieving high-quality research related to RB. However, their research was confined to a certain country or was conducted earlier and did not pay attention to the new trends in RB research. Looking at a longer time horizon, our research can show different stages and turning points in the development of research in this field. This will inform the direction of future research in this area and the formulation of health policy. Our bibliometrics research spans 44 years and highlights not only what has been fully studied, but also what has not been fully explored. We are concerned that the number of research papers on topics such as “liquid biopsies” or “genetic biomarkers” for RB has surged in recent years, suggesting that these lines of research are on the rise and worthy of attention and funding in the coming years.

There are several limitations to this study. Because we only included literature in English, many important research papers published in other languages were not included. Owing to the limitation of the research time frame, many newly published studies have not accumulated many citations, which affected our results to some extent.

## Conclusion

This study describes global trends in RB research. The United States leads the world in the number of RB research papers, followed by China, India, Canada and Germany. Thomas Jefferson University was the most published research institution. Major research papers are available in *Invest Ophth Vis Sci*, *British Journal of Ophthalmology* and *Ophthalmology*. Abramson DH, Shield CL and Francis JH are important collaborators in this direction. RB’s research focus has changed in the last 40 years. Liquid biopsy, intra-arterial and intravitreal chemotherapy are potential hotspots for future RB research.

## Data Availability

The original contributions presented in the study are included in the article/supplementary material. Further inquiries can be directed to the corresponding author.
